# In Vivo Cannulation Methods for Cardiomyocytes Isolation from Heart Disease Models

**DOI:** 10.1371/journal.pone.0160605

**Published:** 2016-08-08

**Authors:** Zhong Jian, Yi-Je Chen, Rafael Shimkunas, Yuwen Jian, Mark Jaradeh, Karen Chavez, Nipavan Chiamvimonvat, Jil C. Tardiff, Leighton T. Izu, Robert S. Ross, Ye Chen-Izu

**Affiliations:** 1 Department of Pharmacology, University of California Davis, Davis, California, United States of America; 2 Microsurgery Core of Department of Pharmacology, University of California Davis, Davis, California, United States of America; 3 Department of Internal Medicine, Division of Cardiology, University of California Davis, Davis, California, United States of America; 4 Department of Medicine and Cellular & Molecular Medicine, University of Arizona, Tucson, Arizona, United States of America; 5 Veterans Administration Healthcare System, Cardiology Section, University of California San Diego, San Diego, California, United States of America; 6 Department of Biomedical Engineering, University of California Davis, Davis, California, United States of America; University of Cincinnati College of Medicine, UNITED STATES

## Abstract

Isolation of high quality cardiomyocytes is critically important for achieving successful experiments in many cellular and molecular cardiology studies. Methods for isolating cardiomyocytes from the murine heart generally are time-sensitive and experience-dependent, and often fail to produce high quality cells. Major technical difficulties can be related to the surgical procedures needed to explant the heart and to cannulate the vessel to mount onto the Langendorff system before in vitro reperfusion can begin. During this period, transient hypoxia and ischemia may damage the heart, resulting in low yield and poor quality of cells, especially for heart disease models that have fragile cells. We have developed novel in vivo cannulation methods to minimize hypoxia and ischemia, and fine-tuned the entire protocol to produce high quality ventricular myocytes. The high cell quality has been confirmed using important structural and functional criteria such as morphology, t-tubule structure, action potential morphology, Ca^2+^ signaling, responsiveness to beta-adrenergic agonist, and ability to have robust contraction under mechanically loaded condition. Together these assessments show the preservation of the cardiac excitation–contraction machinery in cells isolated using this technique. The in vivo cannulation method enables consistent isolation of high-quality cardiomyocytes, even from heart disease models that were notoriously difficult for cell isolation using traditional methods.

## Introduction

Isolation of high quality cardiomyocytes is critically important for achieving successful and reproducible experiments in cellular and molecular cardiology. However, a majority of the methods used for isolating cardiomyocytes from murine hearts are known to be technically challenging and often produce variable results, especially for some heart disease and transgenic models that have particularly fragile cardiomyocytes. The traditional method of isolating cardiomyocytes uses a series of procedures that are critically time sensitive and highly dependent on experience [1–6]. Generally in the traditional approach, the chest is opened which causes cessation of respiration and therefore subjects the heart and its cells to a hypoxic environment. Next, the heart is excised; the blood is washed away, and the excess tissue removed to expose the aorta. Subsequently, the small caliber aorta must be cannulated to allow its attachment to the Langendorff system; and only then, can in vitro perfusion be started to reoxygenate the tissue. These operations usually take a lengthy period of time during which transient hypoxia and ischemia can damage the heart, resulting in a low yield and poor quality of cardiomyocytes. Moreover, as a common practice butanedione monoxime (BDM) is often used to eliminate the contraction of myofilaments during the procedure[7] in order to decrease reoxygenation injury[8] and potentially protect hearts from ischemia–reperfusion injury[7–9]. However, BDM is a non-specific phosphatase and can cause significant side effects on the cardiomyocytes including a loss of excitability[3], altered electrical properties and abnormal Ca^2+^ handling[10–13]. Therefore it remains challenging to isolate high quality cardiomyocytes from murine hearts that are free from artifacts associated with non-physiological cell isolation conditions.

Preservation of an adequate energy supply is among the most critical of factors to allow functional recovery of the cardiomyocytes after the isolation process[14, 15]. We, like others, have noticed that one major factor behind failed cell isolations is the duration of ischemia. Most importantly, many heart disease models are extremely sensitive to ischemia, more so than wild-type control hearts. The ATP consumption is increased significantly in some genetically-manipulated heart disease models[16–19], which may cause these hearts to be more vulnerable to ischemic injury than the healthy control. The ultimate challenge for some of our experiments is to isolate cardiomyocytes that can perform well in conditions of repetitive excitation-contraction under mechanical load. Typically this demands an even greater energy supply be available than load-free conditions.

Here we describe a new cell isolation method using in vivo cannulation of the carotid artery or the aorta, in conjunction with cardiac arrest, to minimize myocardial ischemia. This fine-tuned but easily reproducible protocol has enabled us to consistently obtain high-quality cardiomyocytes from murine wild-type and heart disease models, to a greater extent than other protocols our laboratory has used in the past. The purpose of this paper is to make available this novel method for isolating high quality cardiomyocytes to a range of experimentalists. We illustrate these concepts by measuring the ability of the single cardiomyocytes to perform steady-state excitation, Ca^2+^ signaling, and mechanical contraction; to respond to beta-adrenergic stimulation, and to contract robustly under mechanical loads. This methodology should be useful for many investigators.

## Materials and Methods

### Equipment

A Langendorff system for constant retrograde perfusion and pressure measurement of murine hearts was set up as described previously[6]. The perfusate in the reservoir was pumped into the heart via a cannula onto which the aorta or carotid artery was mounted. The temperature of the perfusate was maintained by pre-heating it in a water bath, and also by a heating coil that wraps around the perfusion tubing. The temperature of the perfusate delivered from the cannula was calibrated to 37°C by adjusting both the heating bath and the heating coil. A 3-way valve was interposed between the outlet of the heating coil and the vessel cannula, with a pressure transducer (BLPR2, WPI) mounted on the third line, so that pressure changes displayed on a pressure monitor (SYS-BP1, WPI) could be monitored during the perfusion and digestion procedure. A basic microsurgery platform was set up for in vivo cannulation and for monitoring the heart tissue during enzyme digestion.

### Solutions

All solutions were freshly made and sterile-filtered using a 0.22 μm filter, right before use. Practical instructions are detailed below for making the various solutions used in this protocol.

1. *Perfusion solution base* (*in mmol/L*) contained: NaCl 113, KCl 4.7, MgSO_4_ 1.2, Na_2_HPO_4_ 0.6, KH_2_PO4 0.6, NaHCO_3_ 12, KHCO_3_ 10, Taurine 30, Hepes 10 and glucose 5; pH = 7.4 (adjusted with NaOH) and Osmolality = 302 mOsm.
2. *Cardioplegic solution* was made by using 100 ml *perfusion solution base* and adding 1mg/ml bovine serum albumin (BSA). This Ca^2+^ free and high K^+^ (15.3 mmol/L total K^+^ in *perfusion solution base*) is used for arresting excitation-contraction.
3. *Enzyme digestion solution* was made by using 50 ml *perfusion solution base* to dissolve 300 u/ml Collagenase type II (Worthington Biochemicals, USA) and 0.04 mg/ml protease type XIV (Sigma, USA), and then adding 12.5 μmol/L Ca^2+^. This enzyme solution can be recycled if more digestion time is needed. Note that different batches of Collagenase may have different enzyme activity (specified in the datasheet from Worthington Biochemicals). Hence the optimal enzyme concentration needs to be found by experimental testing. We usually start with the above condition and then adjust the enzyme concentration to find the optimal result empirically.
4. *BCS solution* was made by adding 3 ml bovine calf serum (BCS) into 27 ml *perfusion solution base*, and then adding Ca^2+^ 12.5 μmol/L.
5. *Incubation solution base (in mmol/L)* contained: NaCl 133.5, KCl 4.0, NaH_2_PO_4_ 1.2, HEPES 10, MgSO_4_ 1.2, and glucose 10; pH = 7.4 (adjusted with NaOH) and Osmolality = 298 mOsm.
6. *Ca*^*2+*^ *ladder solutions* were made by distributing 25 ml *Incubation solution base* into each of three test tubes, and then adding 50 μl, 125 μl, and 250 μl of Ca^2+^ stock solution (100 mM) into each tube to make solutions of 0.2 mmol/L, 0.5 mmol/L, and 1.0 mmol/L Ca^2+^ concentration, respectively. The solutions were warmed to 37°C in a water bath.

### Animal preparation

The use of animals in this study was reviewed and approved by the University of California, Davis, Animal Use and Care Committee (IACUC), and conducted in accordance with the guidelines of Animal Use and Care of the National Institutes of Health and the University of California.

Mice (male, 12–18 week of age) were anesthetized using isoflurane with medical grade oxygen. Initially 4% isoflurane was used in an induction chamber and then 1.2%–2% isoflurane was delivered through a nosecone, to maintain deep anesthesia throughout the entire procedure. A toe pinch test was performed initially after induction to make sure deep anesthesia was achieved, before performing any surgical procedure. After shaving the hair and disinfecting the surgical area with 70% ethanol, a small skin incision was made at the left inguinal area to expose the femoral artery, and then heparin (500 IU) was injected to prevent blood coagulation.

### In vivo cannulation methods

#### Carotid artery cannulation (Fig 1A and 1B)

**Fig 1:**
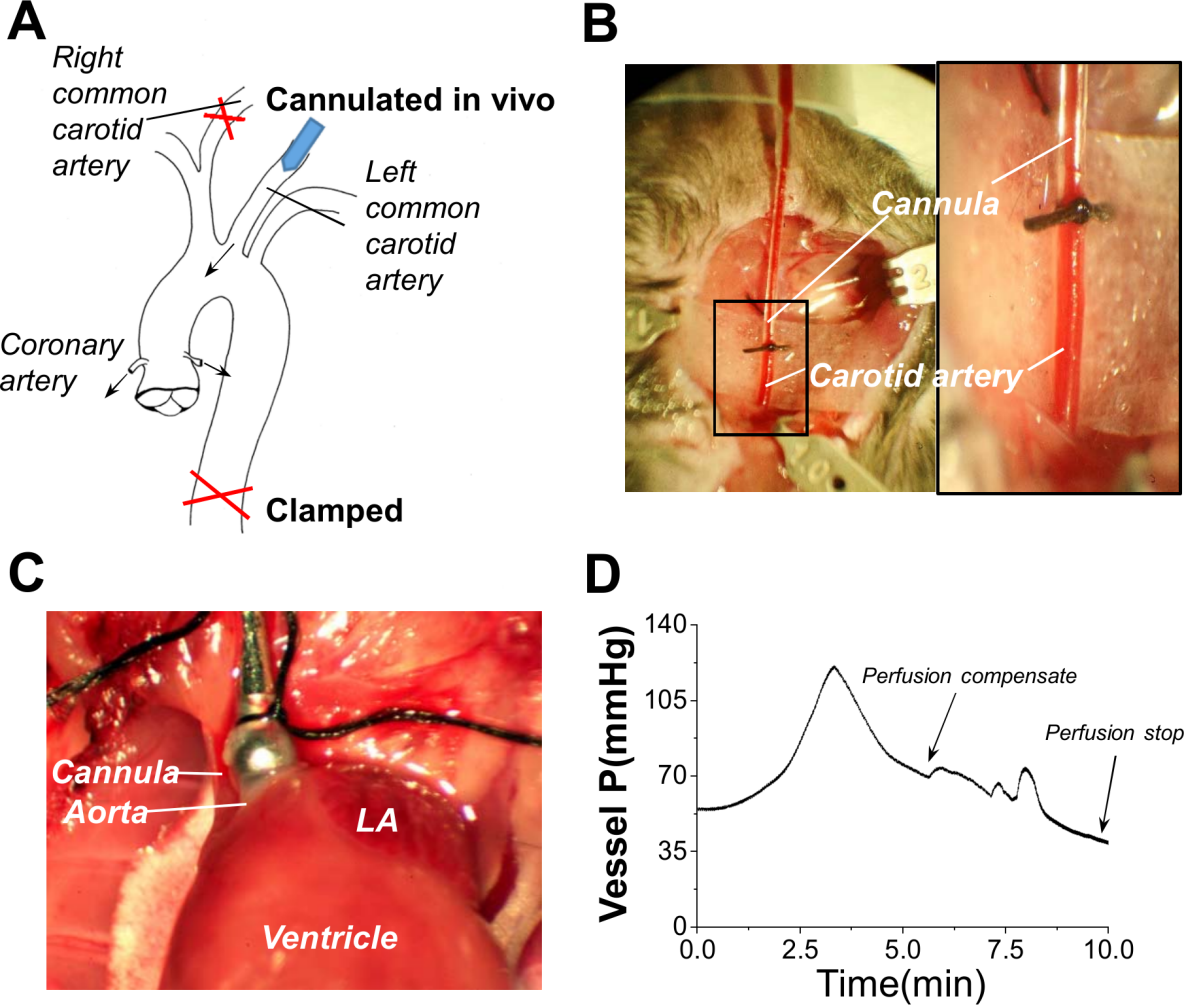
In vivo cannulation methods. (A) Schematic diagram of *in vivo* cannulation via the common carotid artery or via the aorta. (B) shows photos of the carotid artery cannulation with suture of the needle cannula in place. This allow simmediate perfusion of the heart with cardioplegic solution. Illustration of how to clamp the right common carotid artery or descending aorta in place is also shown. (C) shows photo of a simpler procedure used cannulate the aorta. This allows immediate perfusion of the heart. The heart is then excised from the thoracic cavity while maintaining perfusion, hung up onto the Langendorff system. Then the cell isolation procedure is started. (D) shows a representative profile of vessel pressure change during the enzymatic digestion. The pressure usually experiences a transient increase at the beginning of enzymatic digestion. We adjust the perfusion rate to keep pressure lower than 120 mmHg, to avoid aortic valve damage. After this transient increase, the pressure gradually decreases as the enzyme digestion progresses. Continuous compensation of the perfusion rate is used to keep the pressure at 70 mmHg for aortic cannulation, and 90 mmHg for carotid artery cannulation, until the pump rate reaches up to 6 ml/min (arrow). Typically then the vessel pressure suddenly drops. This indicates the appropriate time to terminate enzyme digestion (arrow).

Carotid cannulation was used (instead of aortic cannulation described below) when cardiomyocytes were judged to be prone to damage in some genetically-manipulated and heart disease models. In this case, the right common carotid artery (CCA) was exposed through blunt dissection and a ventral midline neck incision. The digastric, sternomastoid, and omohyoid muscles were retracted using metal hooks. A loose knot (6–0, silk suture) was placed around the CCA. The left CCA was exposed the same way as described above and isolated from the surrounding tissue. The distal end of the left CCA was ligated before the bifurcation of the internal and external carotid arteries. A loose knot was placed around the CCA to allow holding the cannulas for later use. A blood vessel clamp (No.1, Fine Science Tools, Foster City, CA) was placed at the proximal end of the CCA and a small hole was made in the CCA, close to the knot at the distal end of the ligation. A cannula (30G bent blunt needle, 16mm length) with a plastic tube mounted at the tip **(shown in Fig 1B)** was connected to the Langendorff perfusion system and was held by a micromanipulator. Then the plastic tip was inserted into the CCA and secured by tightening the knot of the suture. The clamp was then removed and the pulse was checked (seen as a pulsation in the cannula tubing) to confirm a successful cannulation. The heart was maintained in near normal physiological conditions by perfusion of oxygen-rich perfusate during the above procedure.

Next, celiotomy was performed to expose the vena cava, and 0.1 ml of high concentration KCl (0.5 M) solution was injected via inferior vena cava to quickly arrest the heartbeat, so that ATP consumption could be reduced. Infusion of the *cardioplegic solution* was immediately started to keep pressure at 80–95 mmHg for 3–5 minutes. Meanwhile, the thorax was opened by sternotomy, the pericardium was removed, and an incision was made on the top of the right atrium. The loose knot on the right CCA was tightened and the descending thoracic aorta was clamped to limit the perfusion to the thoracic area.

#### Aorta cannulation (Fig 1C)

For wild-type mice or strains judged to have less vulnerability to ischemic damage, we simplified the cannulation procedure but still were able to maintain high cell quality and yields. The procedures for anesthesia, surgical site preparation and heparin injection were the same as described above. After waiting 3 minutes for heparin distribution, celiotomy was performed to expose the vena cava. Then 0.1 ml of 0.5 M KCl solution was injected to arrest the heartbeat. The thorax was opened by sternotomy and the aorta arch was identified and isolated. A 22G metal cannula (Kent Scientific Co., Torrington, Connecticut, USA) mounted on a 1ml syringe filled with *Cardioplegic solution*, was inserted into the aorta and secured by 6–0 silk suture. After a small incision was made on the top of the right atrium, the heart was reperfused by a syringe injection, to push out blood. Then the heart was excised from the chest and linked to the Langendorff perfusion system. Infusion of the Cardioplegic solution was immediately started at a 3 ml/min rate. Note that it is critical to avoid any air in the perfusion system by starting the solution flow before linking the heart to the cannula in the Langendorff system.

### Enzyme digestion

The *Cardioplegic solution* perfusion rate was adjusted to achieve a stable pressure at 50–70 mmHg for aorta cannulation or 80–95 mmHg for carotid artery cannulation. After this pressure was maintained for 3–5 minutes, the perfusate was switched to the *enzymatic digestion solution*. While the heart was under enzymatic digestion on the Langendorff system with constant perfusion rate, the vessel pressure during the digestion process usually displayed a characteristic profile as shown in **Fig 1D.** Based on this, we developed a criterion for judging the appropriate time to stop enzyme digestion.

As illustrated in **Fig 1D**, during the early phase of enzyme digestion the pressure showed a transient increase. It was generally necessary to adjust the perfusion rate to keep the pressure lower than 120 mmHg to avoid risking aortic valve damage. After the initial transient increase, then the pressure was seen to gradually decrease as the enzyme digestion progressed. During this period, we continuously adjusted the perfusion rate to maintain the pressure at about 70 mmHg for aortic cannulation, and at about 90 mmHg for carotid artery cannulation. These pressure levels were maintained until the pump rate reached 6 ml/minute. Eventually, the vessel pressure gradually decreased. When the pressure dropped to 30 mmHg for aortic cannulation or 50 mmHg for carotid artery cannulation, we terminated enzyme digestion.

Successful completion of enzyme digestion was determined empirically since the myocardial tissue became soft to the touch and appeared flaccid and pale. In our experience, the duration of enzymatic digestion varied for different animal strains and also for different batches of enzyme. (Collagenase can have different activity levels, ranging from 215–305 u/mg.) For example, the digestion times varied from 6.5–12 minutes for wild type C57Bl/J6 mouse hearts, 7–11 minutes for *mdx* (dystrophin-null) hearts, and 12–15 minutes for the familial hypertrophic cardiomyopathy model FHC (cTnT-R92Q) and vinculin heterozygous knockout (*vcl*^*+/-*^). We found that once a unique perfusion pressure profile was determined to indicate appropriate enzymatic digestion of a particular strain, it would consistently produce high quality cardiomyocytes from that strain.

In our experience, the *aortic cannulation* method produced improved cell quality compared to the conventional method, for all murine heart models tested. This included C57BL/6 wild-type and various transgenic mouse models including *nNOS*^*-/-*^, *eNOS*^*-/-*^, and *mdx*. However, the *carotid artery cannulation* method is needed for isolating high quality cardiomyocytes from FHC and *vcl*^*+/-*^ models which are notoriously difficult with the traditional method.

### Cell isolation and Ca^2+^ ladder

For the final part of the protocol, the heart is cut down from the cannula and transferred to a 35mm petri dish containing *BCS solution*. Use a pair of sharp, fine scissors to dissect the heart; ventricular tissue was cut into 4–5 small sections which were then gently teased apart with fine-tip forceps. The tissue was then gently triturated with a transfer pipette to release cells from the tissue. For some transgenic and disease models known to have fragile cells, such as FHC or *vcl*^*+/-*^, shaking trituration (20mins, 60rpm, Reciprocal shaking bath 2870, Thermo Scientific, Waltham, Massachusetts, USA) was used instead of pipetting. The cell suspension was filtered through a 250 μm mesh to separate cells from remaining tissue pieces. Then the ventricular myocytes were suspended in BCS solution containing 12.5 μmol/L Ca^2+^.

Next, we use the *Ca*^*2+*^ *ladder solutions* to gradually increase the Ca^2+^ concentration towards a physiological level. The cardiomyocytes were collected from CBS solution by allowing cells to sink to the bottom, then removing the supernatant, and resuspending the cells in the *Ca*^*2+*^ *ladder solutions* in three steps, by successive addition of 0.2 mM Ca^2+^ (20 minutes), then 0.5 mM Ca^2+^ (15 minutes), and finally 1.0 mM Ca^2+^ (15 minutes). The cells were then stored in the solution with 1.0 mM Ca^2+^ at room temperature (18–22°C) for further use.

### Fine-tuning of the protocol for various transgenic and heart disease models

We have described a basic protocol for using in vivo cannulation to isolate high quality cardiomyocytes. This is summarized in Table 1. The basic protocol can be fine-tuned to optimize the conditions for different strains of mice. For example, we have found that the wild type C57Bl/J6 (WT), eNOS^-/- [^^20^^–^^22^^]^, nNOS^-/-[^^23^^–^^25^^]^, mdx[26, 27], and mice subjected to transverse aortic constriction (TAC)[28], can yield high quality cells by using aorta cannulation. However, in our experience, cardiac myocytes obtained from models such as familial hypertrophic cardiomyopathy model FHC (cTnT-R92Q) and vinculin heterozygous knockout (vcl+/-)[29–32] are more fragile and reproducible successful isolation required the carotid artery cannulation method, adding 12.5 μM Ca^2+^ in enzyme digestion solution, and using shaking trituration to gently separate cells from digested tissue. In our experience, such fine-tuning has been necessary for isolating viable cells from models such as the FHC and vcl^**+/-**^ hearts, where sarcolemmal or sarcomeric proteins may predispose the cells to damage during the isolation process. This fine tuning was absolutely required for these types of models so that cells could be used for functional study of their excitation-contraction capacities, particularly under mechanically loaded condition.

**Table 1:** Basic protocol for cardiomyocyte isolation using in vivo cannulation.

Cardiomyocyte Isolation Protocol Step-by-Step Flow Chart	Aorta Cannulation	Carotid Artery Cannulation
Pre-sedation using isoflurane 5% + O_2_ in an induction chamber	√	√
Anesthesia using isoflurane 1.2–2% + O_2_ via nose cone	√	√
Heparin injection into the femoral artery	0.1 ml, 500 u	0.1 ml, 500 u
Arrest heart by injecting *KCl solution* via vena cava	0.1 ml, 0.5 M	0.1 ml, 0.5 M
**In vivo cannulation**	aorta	carotid artery
Infusion of *cardioplegic solution*, at 37°C	√	√
Infusion of *enzyme digestion solution*, at 37°C	√	√
Stop enzymatic digestion (determined by monitoring the pressure profile)	7–12 min	10–15 min
Dissect out the ventricular tissue	√	√
Isolation of cardiomyocytes by trituration in petri dish	pipetting	shaking
Stop enzyme digestion by incubating cells in *BCS solution*	15 min	15 min
Progressive increase of Ca^2+^ using Ca^2+^ *ladder solutions*	√	√
Store cells in *incubation solution with 1 mM* Ca^2+^, at 18–22°C	√	√

### Confocal imaging of Ca^2+^ transients and cell morphology

As described in our previous publication[33], freshly isolated myocytes were loaded with the Ca^2+^ indicator dye Fluo-4/AM, and paced by the bipolar electrical field stimulation with short (4 ms) depolarization pulses at 0.5Hz.The acquisition of fluorescence images used an Olympus FluoView 1000 Confocal system (Center Valley, Pennsylvania, USA), in which an inverted microscope with a water immersion fluorescence objective (UPlanSApo 60X, NA1.2) was used. Fluo-4 AM was excited with a 488 nm laser beam (laser power set to 5%), and the emission was filtered by a bandpass filter BA505-605.

### Fura-2 measurement of whole cell Ca^2+^ transient

Fura-2 loading was done by incubating the cells with 2.5 μM Fura-2/AM and 0.75 μM Pluronic-127 (in 20% DMSO) at room temperature for 30 minutes, followed by 45 minutes incubation. The dye loaded cells were perfused in Tyrode solution containing (in mM): 150 NaCl, 5.4 KCl, 1.2MgCl2, 1 CaCl2, 10 Glucose at pH 7.4 during the experiment. An IonOptix system with a Hyperswitch (IonOptix Inc., USA) and a Olympus X71 inverted microscope with a water immersion fluorescence objective UPlanSPao 40X, 1.15 NA (Olympus USA), were used for fluorescence excitation (340 nm and 380 nm) and emission acquisition (510/40nm filter) by photomultiplier tube (PMT).

### Measurement of myocyte contraction

The images of contracting myocytes were recorded using a high speed camera (Myocam-S, 240 up to 1000 frame/s, IonOptix system). The sarcomere pattern along the longitudinal axis was used to calculate the sarcomere length during myocyte contraction in real time, by using a Fast Fourier Transform algorithm. The myocyte contraction was measured by the shortening of sarcomere length.

## Results

Using the in vivo cannulation methods, we have consistently obtained high yield and high quality cardiomyocytes from both wild-type and heart disease models. Importantly, the in vivo carotid artery cannulation method enabled us to obtain high quality ventricular myocytes from some heart disease models, when this was difficult or impossible with conventional methods. The high quality of cardiomyocytes has been verified by important structural and functional measures.

### Morphology of cardiomyocytes

**Fig 2:**
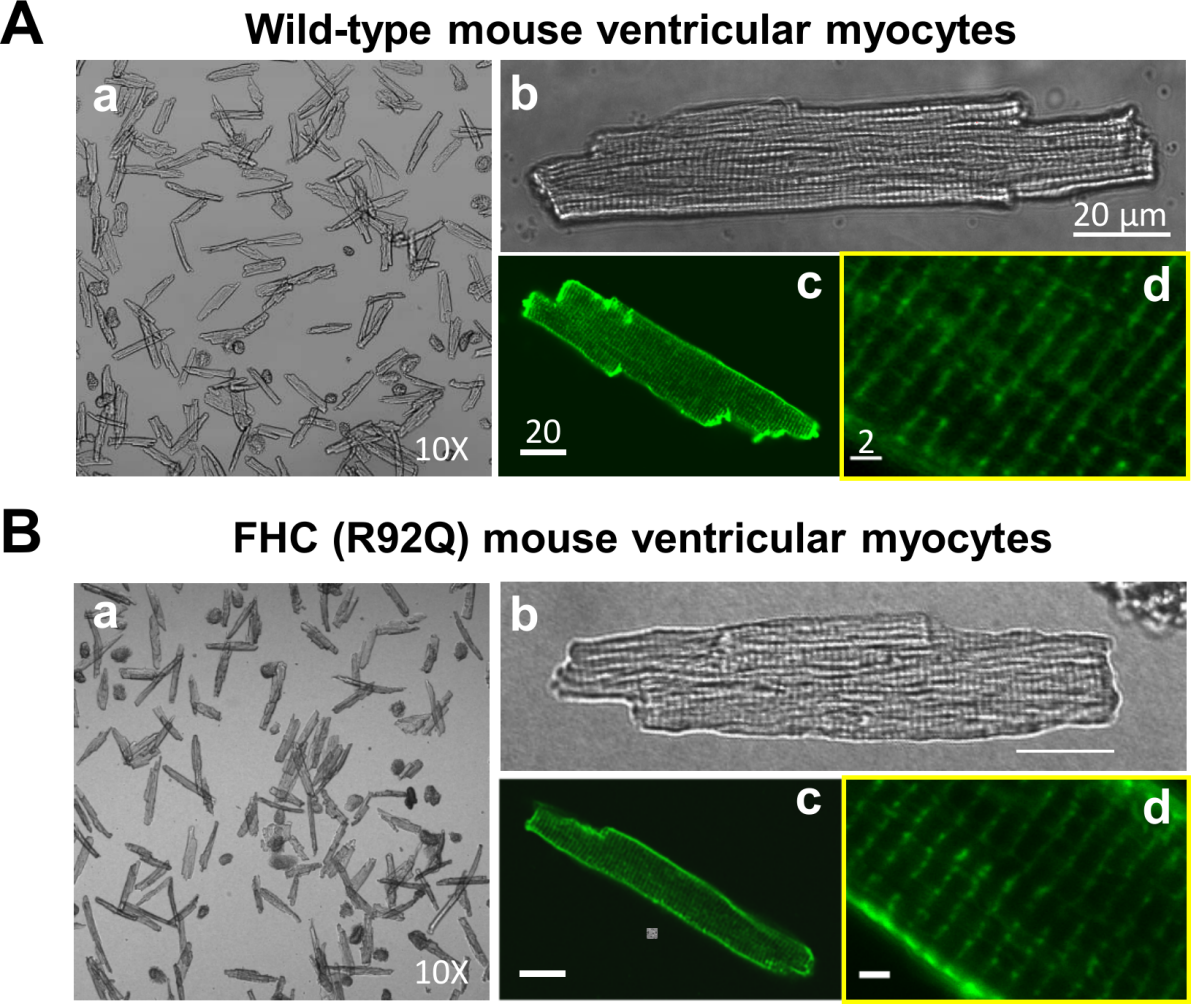
Cardiomyocytes yield, morphology, and t-tubule integrity. (A) and (B) show examples of the ventricular myocytes freshly isolated from a wild-type (WT) C57Bl/J6 mouse heart and from a cardiomyopathy model (FHC, cTnT-R92Q), respectively. The yield of live ventricular myocytes was about 85% for WT (Aa) and 60% for FHC mice (Ba) as judged by rod-shaped cells; the dead cells appear pop-corn like. The health ventricular myocyte morphology shows rod-shaped cells with defined edges and clear striations showing the sarcomeres (Ab, Bb; scale bar: 20 μm). Di-8ANEPPS fluorescence images show a well-structured T-tubule system (Ac, Bc scale bar: 20 μm). The sarcomere length (SL) measured from the transmittal images and from the Di-8 fluorescence images (Ad, Bd; scale bar: 2 μm) show an average slack SL value of 1.8 μm.

**Fig 2A** shows an example of wild-type ventricular myocytes isolated using the aortic cannulation method. **Fig 2B** shows ventricular myocytes isolated from FHC model using the carotid artery cannulation method, which exemplifies the high quality of cardiomyocytes isolated from this most difficult model. In all cases, the ventricular myocytes typically showed about 90% rod-shaped, viable cells, versus only 10% appearing as “popcorn-shaped” dead cells. The morphology of the ventricular myocytes showed clear striations, sharp edges, and a clean surface. Di8-ANEPPS staining showed clear and registered t-tubule structure (**Fig 2C and 2D**). This excellent preservation of cell morphology was maintained during the entire course of our experiments, for approximately 6–8 hours after cell isolation.

### Excitation, Ca^2+^ signaling, and Contraction

**Fig 3:**
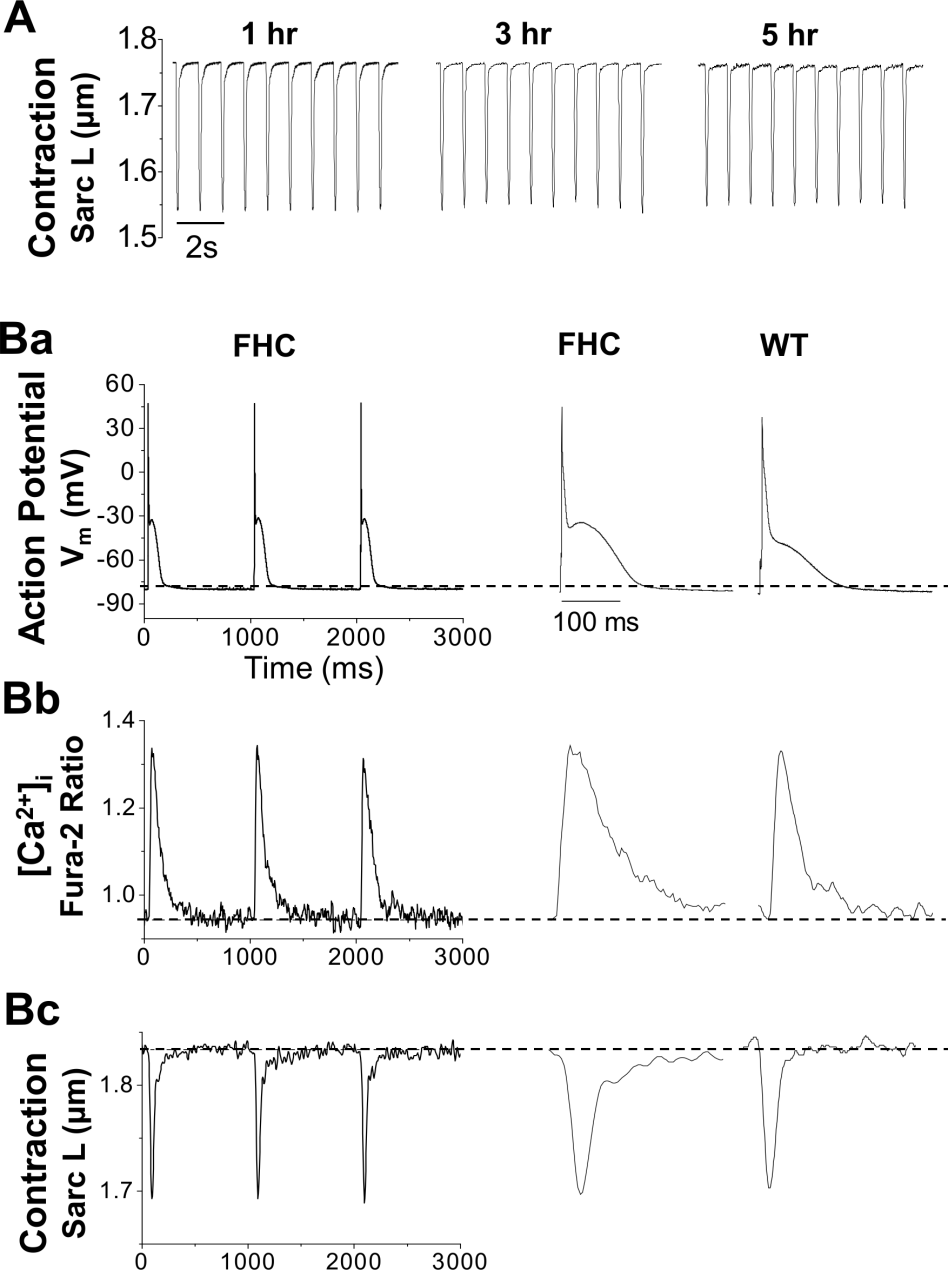
Functional study of the AP, [Ca^2+^]_i_ and contraction of isolated cardiomyocytes. Functional studies of the excitation-contraction coupling properties were performed on WT and FHC cardiomyocytes paced at 1.0 Hz, at body temperature (37°C). (A) Long-term monitoring of cell contraction. The cells showed robustness of sarcomere length and fractional shortening up to at least 5 hours after isolation. (B) shows typical recordings of the action potential (Ba), the [Ca^2+^]_i_ measured using fura-2 fluorescence ratio (Bb), and the contraction measured by shortening of the sarcomere length (Bc). All three signals reach steady state, indicating the high quality of cells. FHC showed a longer APD, slower Ca^2+^ transient and slower contraction than the wild-type.

**Fig 3A** shows that the isolated cardiomyocytes can achieve robust steady-state contraction, measured as sarcomere shortening, at 1, 3, and even 5 hours after cell isolation. Normally the cells were used for experiments within 6–8 hours. Furthermore, we assessed the excitation-contraction characteristics of cardiomyocytes by monitoring the action potential, the Ca^2+^ signal, and the mechanical contraction during pacing. **Fig 3B** shows the simultaneous recording of these three signals. The high quality of the cells was demonstrated by their ability to reach steady-state action potential, Ca^2+^ transient, and contraction during repetitive pacing (**Fig 3**Ba). Compared to wild-type cells (**Fig 3**Bb, WT), FHC cells showed prolonged action potential, slower Ca^2+^ transient, and slower contraction (**Fig 3**Bb, R92Q).

### Responsiveness to beta-adrenergic stimulation

**Fig 4:**
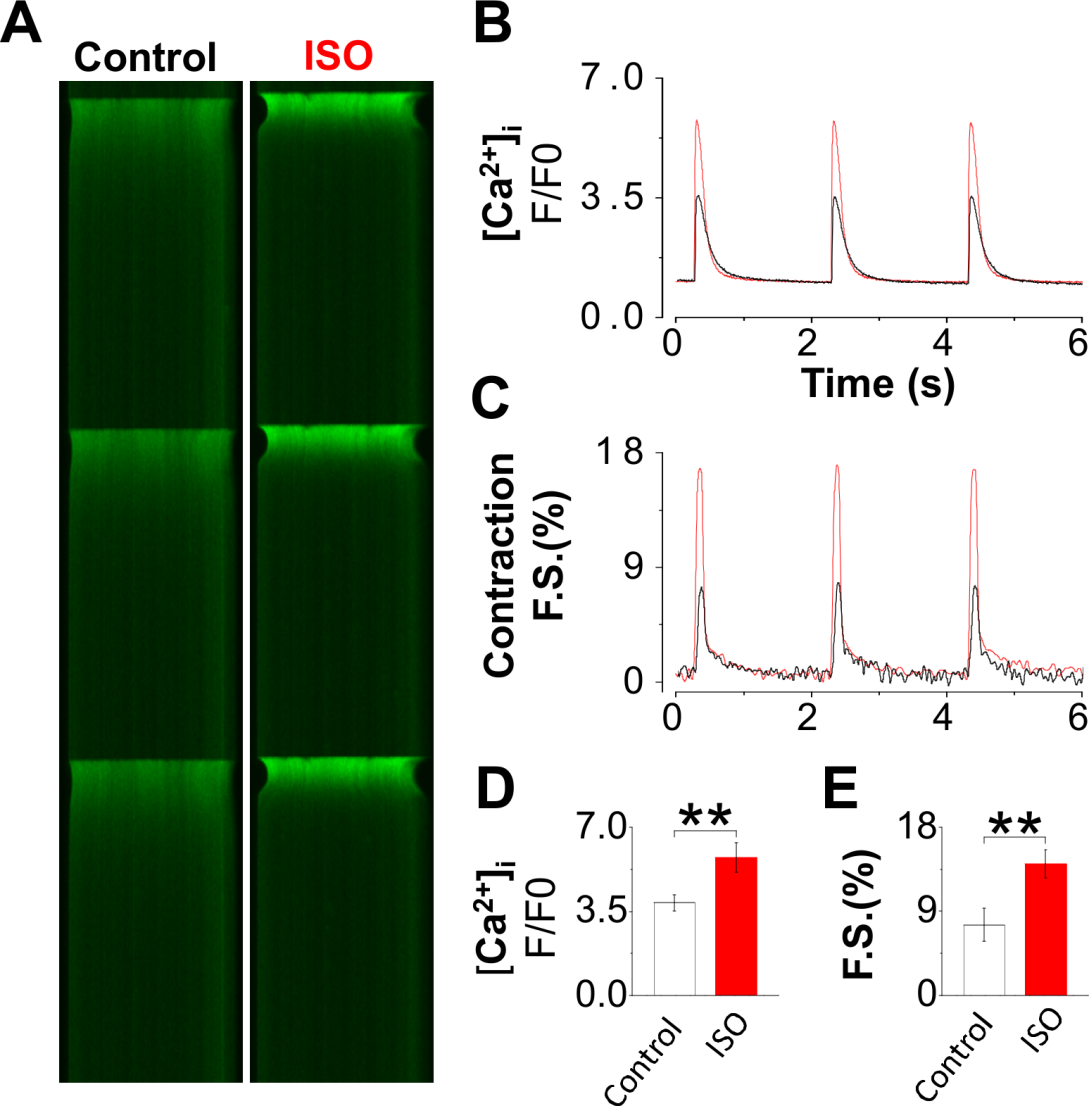
Response to beta-adrenergic stimulation of cardiomyocytes from R92Q. The responsiveness of cardiomyocytes to beta-adrenergic stimulation was tested using 30 nM isopreterenol (ISO). The myocytes were paced at 0.5 Hz at room temperature (21°C). The Ca^2+^ signal and cell contraction were recorded using Fluo-4 fluorescence confocal microscopy (A). Cardiomyocytes contracting in normal Tyrode solution were defined as the load-free condition. Confocal linescan images show ISO treatment markedly increased the Ca^2+^ transient measured by Fluo-4 fluorescence F/F_0_ ratio (B, D, n = 7 for control and ISO applied group). and enhanced the load-free contraction measured as fractional shortening (C, E, n = 7 for both groups). Unpaired Student’s t test; *P < 0.05, ***P < 0.001.

Preservation of cell-surface receptors is an important criterion for high quality cell isolation. To assess this we evaluated the responsiveness of the cardiomyocytes to beta-adrenergic receptor stimulation. **Fig 4A** shows that the FHC cells are responsive to isoproterenol (ISO). The cells were also able to reach steady-state excitation-contraction under beta-adrenergic stimulation. ISO increased the Ca^2+^ transient (**Fig 4B and 4D)** and the contraction of cells (**Fig 4C and 4E)**, demonstrating intact functional beta-adrenergic receptors and signaling pathways. Similarly, the wild-type cardiomyocytes were able to respond to ISO and reach steady-state excitation and contraction. These results demonstrate that the cell-surface beta-adrenergic receptors are well preserved after cell isolation and enzymatic digestion, even in FHC cells that are more vulnerable to the cell isolation procedure than other strains.

### Cardiomyocytes contraction under mechanically loaded condition

**Fig 5:**
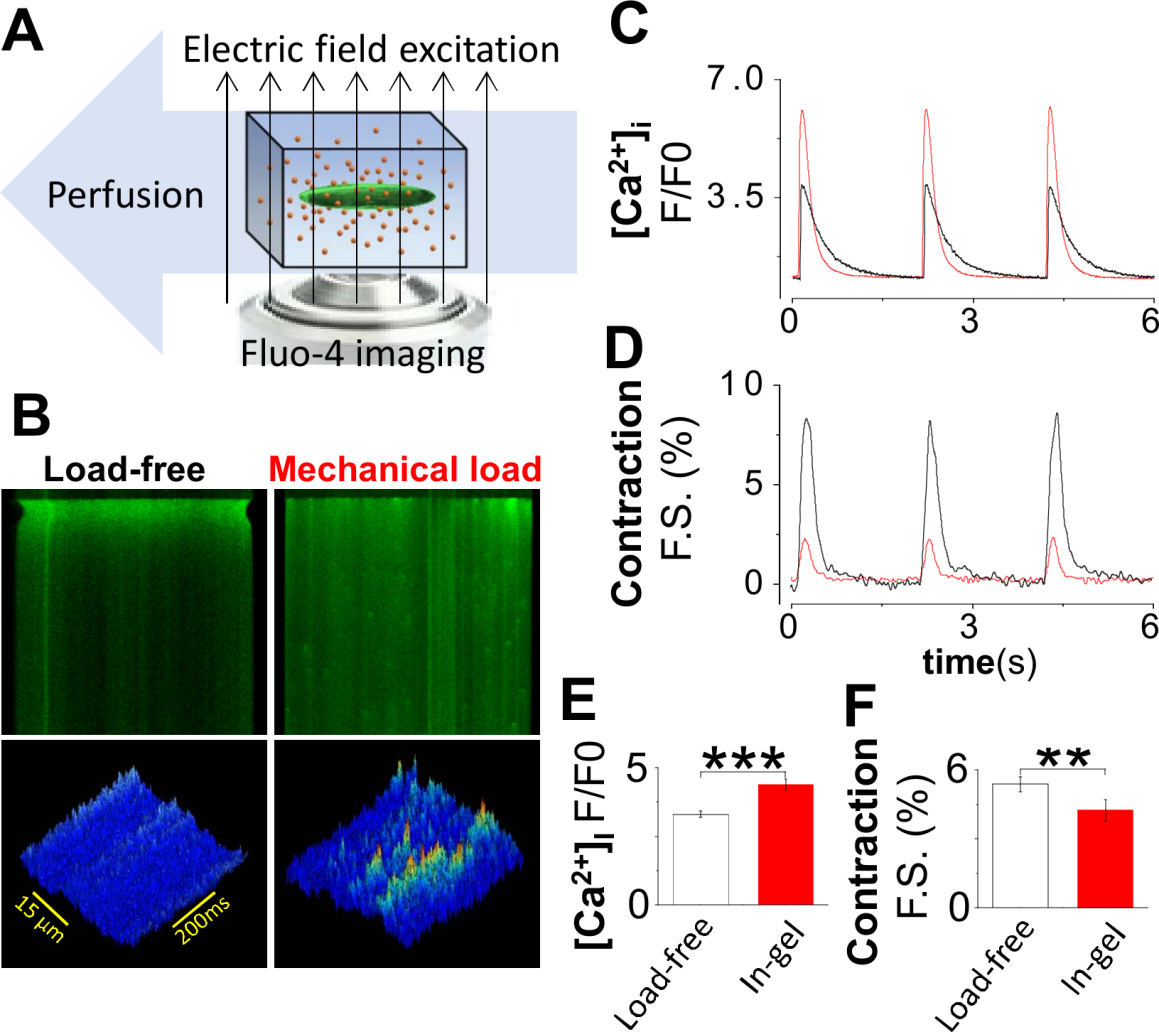
Mechanically loaded contraction and mechano-chemo-transduction of cardiomyocytes from R92Q. (A) We used our Cell-in-Gel system [33] to impose a mechanical load during cardiomyocyte contraction. (B) Fluo-4 confocal linescan images show Ca^2+^ signals during systole and diastole. Cell contraction was seen in the edge shortening. The cells were able to reach steady-state contraction. Cardiomyocytes contracting in normal Tyrode solution were defined as the load-free control. (C) Systolic Ca^2+^ transient (CaT) peak in cell-in-gel (n = 15) vs load-free control (n = 19). (D) Fractional shortening of cardiomyocyte contraction in-gel (n = 15 cells) vs load-free control (n = 18). (E) Mechano-chemo-transduction was seen in the significant enhancement of the Ca^2+^ transient under load (n = 15), compared with load-free group (n = 19). (F) Meanwhile, the fractional shortening was significantly decreased under mechanical load (n = 15), compared with load-free group (n = 18). Unpaired Student’s t test; *P < 0.05, ***P < 0.001.

In vivo, during each heartbeat cardiomyocytes contract under *mechanically loaded condition*. However, most in vitro experiments are conducted using cells in *load-free* conditions that are non-physiological. Recently we developed a Cell-in-Gel method **(Fig 5A)** to impose mechanical load on single cardiomyocytes during contraction. This is necessary for studying the mechanical stress effects and mechano-chemo-transduction in cardiomyocytes[33]. Mechanical loading puts a higher requirement on the energy demand and the overall cell quality than load-free conditions. **Fig 5B** demonstrates that the FHC cardiomyocytes were able to contract in this system under mechanical load. The mechanical loading caused an increase of the systolic Ca^2+^ transient through mechano-chemo-transduction (**Fig 5C and 5E**), and also a reduction of contraction amplitude occurred due to increased afterload (**Fig 5D and 5F**). Similar results were obtained in wile-type cardiomyocytes[33]. In our experience, the in vivo *cannulation* technique is critically important for isolating high quality cardiomyocytes that can contract robustly under mechanical loads.

## Discussion

We have developed a novel and adaptable method to isolate high-quality cardiac myocytes from mouse heart disease models. The innovations of this method include: (1) Use of in vivo cannulation to minimize transient hypoxia and ischemia; carotid artery cannulation is used for transgenic and heart disease models that seem to be “fragile” as compared to aortic cannulation for healthier models. (2) Arrest of heartbeat by injecting KCl solution via the inferior vena cava prior to thoracotomy in order to reduce energy demands and contraction-related injury. (3) Exclusion of butanedione monoxime (BDM) to avoid side effects on myocytes function. (4) Development of standard criterion using the vessel pressure profile during enzyme perfusion, to allow real-time monitoring of the tissue digestion stage, and to determine the optimal time to terminate enzyme digestion. We have combined these innovations into an in vivo cannulation protocol that can be fine-tuned to achieve consistent high-quality cardiomyocytes isolation from mouse hearts of various transgenic and heart disease models.

**Fig 6:**
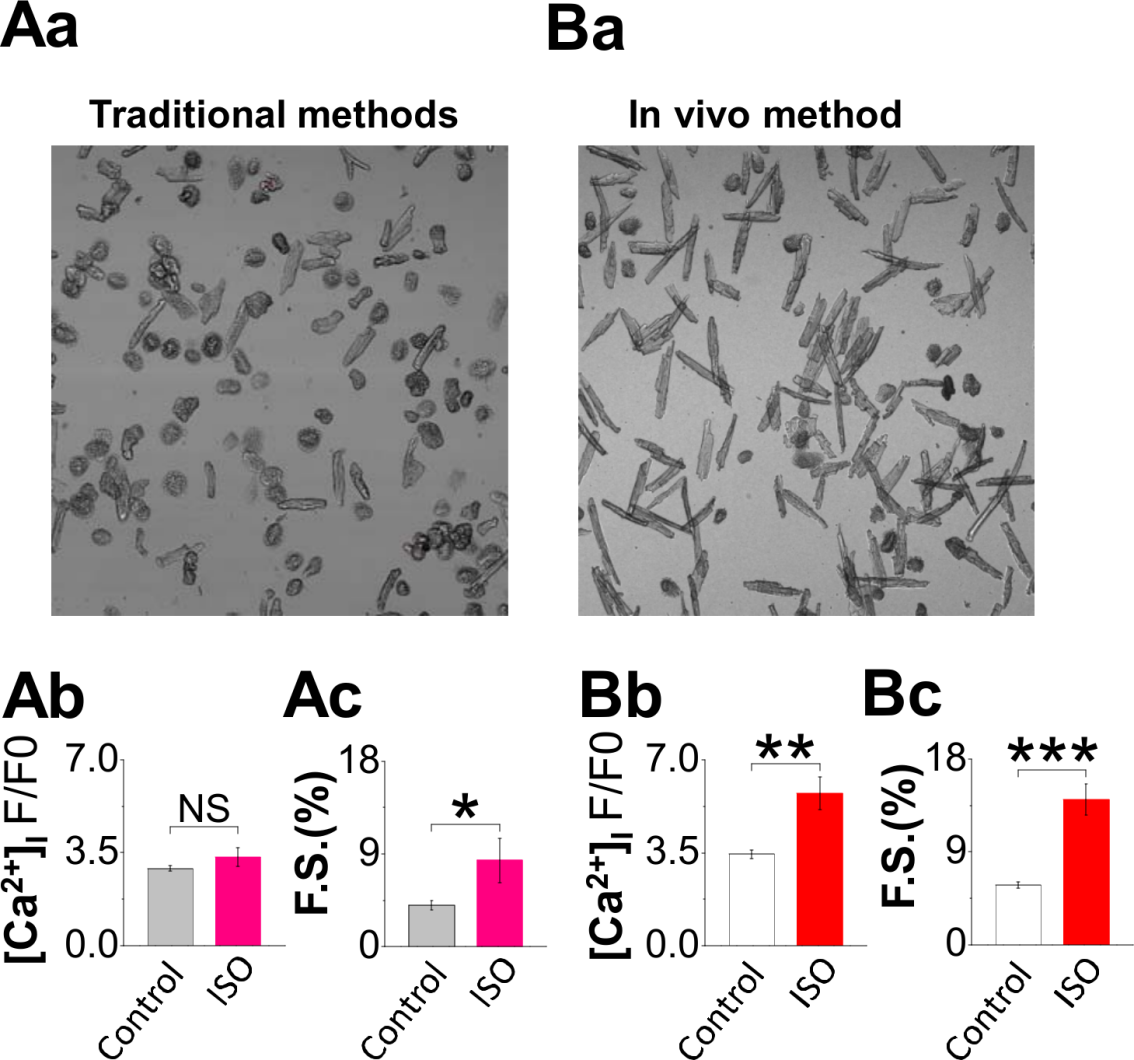
Comparing the cells isolated using traditional method and the in-vivo cannulation method. (A) and (B) show the significant difference of isolation quality of the ventricular myocytes from a cardiomyopathy model (FHC, cTnT-R92Q). The ventricular myocytes were freshly isolated using traditional methods versus using our in vivo cannulation method. The butanedione monoxime (BDM) was excluded from solution recipes because of its significant side effects on the cardiomyocytes. The yield of live ventricular myocytes was normally less than 30% for traditional method (Aa) and larger than 60% for in vivo method (Ba) as judged by rod-shaped cells; the dead cells appear pop-corn like. The healthy ventricular myocyte morphology shows rod-shaped cells with defined edges and clear striations showing the sarcomeres. Compared with cells by in vivo cannulation method, the ventricular myocytes isolated by conventional way showed a smaller Ca^2+^ transient (Ab n = 29 vs Bb n = 19), weaker contraction (Ac n = 24 vs Bc n = 18) and lack of responsiveness to beta-adrenergic stimulation (n = 5 using traditional method vs n = 7 using in-vivo cannulation method). Unpaired Student’s t test; *P < 0.05, ***P < 0.001.

Isolated single cardiomyocytes have been used in a wide range of experiments for studying heart diseases, especially as most studies pursue the cellular and molecular mechanisms underlying the disease process. Many studies require evaluation of isolated live cells that retain function as close to that present when they were in the intact heart. Such mechanistic studies frequently cannot be done at the tissue and organ levels. In particular, functional studies of the excitation-contraction coupling and evaluation of various signaling pathways during cardiomyocyte contraction require high-quality live cells that have preserved all the molecular machinery. Our in vivo cannulation method was indeed developed to meet the stringent requirements for studying live cardiomyocyte functions (i.e. electrical excitation, signaling, contraction under mechanically loaded condition, etc.). In comparison, when we tried using more conventional cell isolation methods with BDM, we found that normal cardiomyocyte excitation-contraction was impaired. BDM was also found to have irreversible effects on cardiomyocytes by other labs[3, 10–13]. Therefore we first opted to avoid use of BDM. But when we tried using the conventional methods without BDM, cell isolation was difficult even for less fragile hearts, and failed almost completely for “fragile” heart disease models. Cell yields were very low (Fig 6), and the few survivors showed smaller Ca^2+^ transient, weaker contraction and lack of responsiveness to beta-adrenergic stimulation. The cells died shortly later and could not be used for further studies.

Because the quality of experimental data critically depends on the cell quality, we made significant efforts to develop the in vivo cannulation method reported here. We also carried out experimental studies to verify preservation of the function of the cardiomyocytes isolated with this new methodology. Importantly, our recently developed Cell-in-Gel technique enabled us to study single cardiomyocyte contraction in a 3-D viscoelastic gel matrix (extracellular matrix surrogate) environment, under mechanically loaded condition (Fig 5). Many previous single cell experiments were performed under less stringent, load-free conditions. In comparison, mechanically loaded contraction puts much higher demands on the cell integrity and energetics than load-free conditions. In our experience, the in vivo cannulation method has been able to consistently produce high quality cardiomyocytes from various mouse heart disease models. This allowed us to meet the new challenges and opportunities of studying single cardiomyocyte function under various mechanical loads mimicking in vivo conditions. We think that the new method reported here will help researchers to isolate higher yields and better quality cardiomyocytes from all types of model systems.
